# West Virginia’s Sugary Drink Tax Examining Print Media Frames in Local News Sources

**DOI:** 10.13023/jah.0102.03

**Published:** 2019-07-06

**Authors:** Lauri A. Andress, Ogaga Urhie, Christine Compton

**Keywords:** Appalachia, sugary drinks, tax, framing effect, obesity, diabetes, media frames, local news services

## Abstract

**Introduction:**

Framing is an important aspect of the policy process that helps the public and decision makers sort through and resolve highly charged claims about an issue. Through slight changes in the presentation of issues, a framing effect may alter public support. The way a proposed sugary drink tax is discussed in public discourse and by the media significantly influences policy acceptance. Given the public health significance of obesity and diabetes in West Virginia (WV) the study of media frames employed to represent a sugary drink tax policy is useful.

**Methods:**

Using quantitative content analysis, this study assessed news articles—published over 7 years by news outlets in WV—to determine the frames that were employed.

**Results:**

Pro-tax arguments appeared more often in these articles. In both pro- and anti-tax arguments, a personal behavior or economic frame appeared more frequently. The more common anti-tax arguments focused on the tax being regressive and not changing personal behavior. The pro-tax arguments focused more often on increases in state revenues and people selecting healthier beverages.

**Implications:**

Given the significance of obesity and diabetes in WV, the study of media frames that represent the sugary drink tax should provide valuable guidance to inform strategies that utilize public discourse and media coverage to influence policy acceptance. However, since WV has not been able to get approval for its sugary drink tax, it may be beneficial to examine other elements of agenda setting including issue generation tactics, mobilizing structures, and political opportunities.

## INTRODUCTION

In the analysis of policy dynamics, framing scholarship lifts up the primacy of cognitive research that examines societal meanings assigned to ideas, narratives, and images.^1^ Arising from existing mental maps, a frame is a socially constructed shorthand expression used to make meaning of phenomena that we encounter.^2^ Moreover, framing research may also examine how the social construction of issues and ideas can be intentionally communicated to encourage a certain definition, causal interpretation, moral appraisal, and/or policy outcome.^3^,^4^ The frames that capture issues are picked up and reflected by the media, which play a major role in shaping and reflecting ideas and views on a given issue.^5^,^6^ The media, in transmitting frames, creates framing effects that occur when transformations in the presentation of an issue or an event produces changes in opinion.^7^ In this way, frames may cancel each other out in a competition between groups for public support.^2^ In this study, newspaper frames used to portray a tax on sugary beverages in West Virginia were identified. Then, because the democratic process links policymaking to the public through the news media, the framing effects that result from competing frames found in the news were considered.

## BACKGROUND

Obesity and adult-onset of type-2 diabetes are major contributors to West Virginia’s poor health rankings.^8^ Driven by such bleak data, the West Virginia American Heart Association (WV–AHA) has worked on passage of a sugary drink tax (SDT) since the fall of 2016. Taxing bottled drinks is not a new idea in West Virginia. An existing container tax, enacted in 1951, is set at 1 cent per 16.9 ounces of every drink sold (regardless of sugar content). When created, this early tax was earmarked to fund the West Virginia University School of Medicine. Notably, this tax was enacted before sugary drinks became a public health concern and continues to be accepted by the soda industry and West Virginia University.

The original legislative proposal incorporating an excise tax of 2-cents per ounce added on at the distributor level (SDT1) remained the same from 2016 to 2017. This tax policy updated a section of the State code to define specific drinks as sugary drinks and imposed an excise tax of 2-cents per ounce added on at the distributor level. The intent from WV–AHA’s perspective was that the tax would be passed along to the consumer, thus driving down purchasing and consumption, eventually resulting in lower rates of diet-related diseases.

Opposition to the 2016 proposal came from the American Beverage Association, convenience store lobbying groups, and retailers associations who, in a manner similar to that found in other SDT campaigns, advanced anti arguments about driving distributors out of business leading to a loss of jobs and the freedom of the consumer to choose the beverage of their choice.^9^–^11^

In 2018 the WV–AHA restructured SDT1 as a “tiered tax” (SDT2). Thought to be more effective in reducing consumption of sugary drinks, supporters of SDT2, also chose to earmark the projected tax revenue to offset state deficit funding for WV–Public Employees’ Insurance Agency. Under the tiered approach beverages were categorized as having high, medium or low sugar content with a different rate of taxation applied to each category. For example, a high sugar beverage, (more than 20g/12oz.), would be taxed at 2-cents per ounce while a drink in the medium tier, (5g–20g/12oz.), would be taxed at 1-cent per ounce. Drinks in the lowest tier, containing less than 5 grams of sugar per 12 ounces would not be taxed at all. Beverages not included under the tax were water (still and sparkling), milk, unsweetened coffee and tea drinks, and 100% juice and diet drinks.

## METHODS

Supplement A (in the Additional Files) provides a detailed description of the research methodology. All the authors were involved in the implementation of a spring 2018 graduate public health course presented at West Virginia University, School of Public Health. The course, Policy Tools for Population Health (Health, Policy Management and Leadership 624), used the SDT as the policy example to examine agenda setting and framing effects.

**Figure 1:**
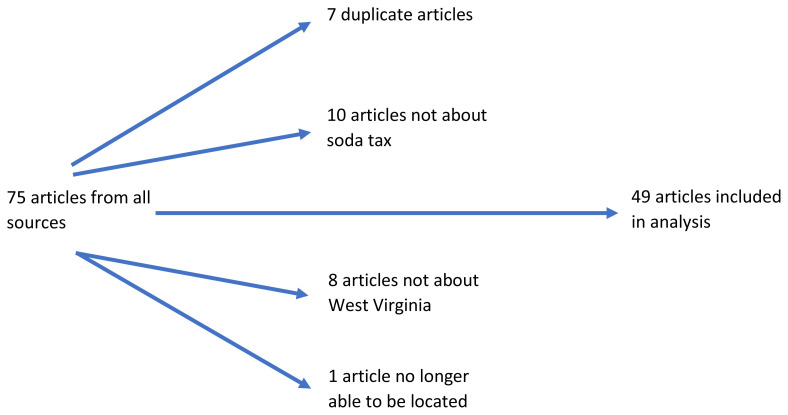
Rationale for articles that were excluded on review of our initial search

This analysis aimed to identify news frames for a sugary drink tax found in West Virginia newspapers from January 1, 2010 to April 10, 2018. This time period was selected to correspond with national sugary drink tax campaigns across the country between 2010 and 2018.^12^ The research methodology was informed by the course materials including case studies, expert interviews, and previous studies demonstrating that the soda industry has typically positioned the SDT as a matter of individual freedom and jobs in previous policy campaigns across the US.^9^,^11^,^13^,^14^ A four-stage coding protocol was developed and applied in order to identify fourteen news sources that were both online and in print, a 49-news article sample, and five major frames (Supplement A, Table 1, Figure 1, in the Additional Files).

- Economics
- Public health concerns
- Personal liberty
- Scientific rationale
- Personal behavior

## RESULTS

### Publication Timeline of Articles

Fewer than five articles were published in any given year between 2010 and 2015. The greatest number of articles published annually was in 2017 (n = 24). A more detailed analysis by month indicated an upward spike in articles published (n= 10) in February 2017. During that same period in 2018 when the tiered tax (SDT2) was introduced there was no similar uptick in articles on the sugary drink tax. See Figures 2A and 2B, Supplement A, in the Additional Files.

### Frames and Arguments

Analysis of the frequency of argument and examples of all arguments from the news articles are in Supplement B in the Additional Files. A total of twenty-one (n=21) different pro- and anti-tax arguments were identified in the news articles sampled (Supplement B, Table 2). The argument found with greatest frequency used an economic frame where 47% of the articles included a claim that the tax would provide revenue to help balance the budget (Table 2). The next most frequently used argument was a personal behavior frame where the claim was made in 39% of the articles that the tax would lead people to choose a substitute beverage. Overall, fewer kinds of anti-tax arguments (n=8) were found, in comparison to pro-tax arguments (n=13) (Tables 3 and 4). Overall, both the pro and anti-tax arguments utilized the economic and personal behavior frames (n=114) more than the other frames all together (n=54). Finally, this analysis of the news article sample indicated that pro-tax arguments (n= 135) were utilized to a greater degree than anti-tax arguments (n=33) in the news articles.

### Economic Frame

Indicating the importance of the economy and employment in West Virginia, the economic frame heightened the issues of cutbacks, reductions, scaling-down, and a declining economy. In total ten types of pro and anti-tax arguments used the economic frame (Table 2). Anti-tax arguments utilized the economic frame (n=13) far fewer times in comparison to the total number of pro-tax arguments made using the economic frame (n=53). The most frequently used anti-tax argument (n=9) with an economic frame focused on the repressiveness of the policy (Table 3). The pro-tax argument used the most (n=23) with an economic frame emphasized the utility of the policy in raising revenue and helping to balance the budget (Table 4).

### Personal Behavior Frame

The personal behavior frame recognizes the primacy of U.S. values around individual accountability for the choices that one makes. This frame is widely recognized as the dominant way that health status is conceptualized in the U.S. as well as other social issues where policies on social assistance are on the agenda.^10^,^15^–^18^ In total four types of pro and anti-tax arguments utilized the personal behavior frame (Table 2). The pro-tax arguments made use of the personal behavior frame (n=37) almost three times more than the anti-tax arguments (n=11). The most frequently used anti-tax argument (n=6) with a personal behavior frame criticized the SDT for changing only the location where people bought their sugary drink as opposed to altering the purchase of the sugary beverage (Table 3). The pro-tax argument used the most (n=19) with a personal behavior frame emphasized how the tax would lead people to select a healthier drink (Table 4).

### Public Health Frame

The public health frame portrayed positivist beliefs where facts and data assume primacy over other constructivist approaches that emphasize the human experience as beneficial in the production of evidence.^19^ In total there were three different pro and anti-tax arguments under the public health frame (Table 2). In comparison to the anti-tax arguments (n=6) under the frame of public health the utilization of pro-tax arguments was greater (n=31). The most frequently used anti-tax argument (n=6) using the public health frame emphasized that SDTs do not address obesity/diabetes (Table 3). The pro-tax argument used the most (n=18) with a public health frame emphasized how the tax would reduce morbidity or mortality from obesity (Table 4).

### Personal Liberty Frame

This frame captures the U.S. value of individual freedom with little or no government infringement of rights. This frame represents the constant struggle between individual freedom versus collective responsibility for social good.^20^ There were two kinds of personal liberty arguments (Table 2). Analysis of the news articles indicated that the pro- and anti-tax arguments used the personal liberty frame equally (n=3). The most frequently used anti-tax argument (n=3) using the personal liberty frame relied on the idea that government was overstepping its boundaries in telling people what to drink (Table 3). The pro-tax argument used the most (n=3) with a personal liberty frame emphasized that government had a role in producing healthy citizens (Table 4).

### Scientific Rationale Frame

The scientific rationale frame defines the issue as a matter of expert understanding and sound science to support or undermine expert consensus.^21^ The analysis indicated that no anti-tax arguments utilized the scientific rationale frame. Both pro-tax arguments using a scientific rationale focused on the effects of sugar on the body where the addictive properties of sugar appeared only a little more frequently (n=6) than the argument about the negative effects of sugar on the body generally (n=5) (Table 4).

## IMPLICATIONS

Framing tactics and trends found in this analysis mirror what has been found in other framing studies on the sugary drink tax where economic and personal behavior frames are used by both supporters and opponents.^11^,^22^,^23^ Across all frames, except for the personal liberty frame, pro-tax arguments appeared in the news articles at a much greater rate than anti-tax arguments. That pro-tax arguments are found more often than anti-tax messages is also consistent with other research.^11^

Generally, arguments in pro- and anti-tax articles used an economic frame. In West Virginia it was easy for proponents or opponents to use the economic frame because arguments about the benefits or harms from the tax could be linked to trends in declining job growth or government revenues.^8^ In this case the anti-tax economic argument emphasized how the sugary drink tax harms jobs. Alternatively, the pro-tax economic argument claimed that the SDT would address revenues and budgetary shortfalls.

The use of an economic frame supporting the sugary drink tax as a positive instrument because it targets “sin taxes” is consistent with other campaigns on products like alcohol or tobacco that promote the potential of the tax to fund positive government expenses such as education.^24^–^27^ Anti-tax arguments using the economic frame made claims most frequently about the SDT as a regressive policy. The impact of this argument works when the opponents to the tax attach the effects of the tax to low wealth groups, and the idea of food as a necessity unlike alcohol and tobacco which are not vital to wellbeing.^26^ The argument is that lower income households would pay a greater proportion of their income in additional taxes compared with higher income earners.^28^

What remains unclear is why the abundance of pro-tax arguments in news articles did not translate into approval of a sugary drink tax by the West Virginia legislature. In fact, despite the dominant use of pro-tax arguments in most regions, the success of the SDT has been variable across the U.S.^14^ Research demonstrates that framing does not constitute the full range of activities needed for agenda setting.^17^,^29^,^30^ In other research an agenda setting framework has been used to determine the strength of issue advocacy efforts by evaluating^1^ how an issue is generated^2^; political opportunities including the nature of the political system and governance issues;^3^ key mobilization resources; and finally^4^ framing strategies.^17^ While emphasis on the message is important, policy advocates must account for the entire playing field including the resources available between groups, building and sustaining carriers of the message, and ensuring a strong physical infrastructure for outreach.^30^ For example, it may be that advocates may have more luck in motivating millennials to support the SDT by generating framing effects similar to the tobacco industry related to social justice, e.g., the big soda company taking advantage of vulnerable groups in Appalachia.^31^,^32^ Most likely the inconsistency in the passage of the SDT in West Virginia is due not only to the framing wars but also the combination of other agenda setting factors.

This research is only one illustration and does not claim to explain causation between the success of SDT legislation and the framing of the tax. It is limited by its singular focus on newspaper articles in West Virginia to the exclusion of other communications strategies including television and radio commercials, billboards, advocacy letters, online postings, and the tactics of lobbyists. We realize there are many different communications tools and sources available from which to secure information. This preference is further segmented by social status. This study tried to address this limitation by selecting articles from newspapers that had a print edition along with an online presence.

## Supplement A

### Four Stage Coding Protocol, Publication Timeline of Articles

#### Stage 1: Selecting news sources

Databases used to search for news articles had to be part of the West Virginia University library system and provide online access. The following databases were used: Google, Newsbank, Newspaper Source, and Proquest. The search of the database yielded fourteen news sources that appeared in both online and print formats (Table 1).

#### Stage 2: Applying inclusion criteria to identify news articles

Key word searches were conducted within the fourteen print online news outlets using two phrases: (1) sugary tax West Virginia and (2) soda tax West Virginia. These words could be in the headline or within the body of a news article. Articles published between January 1, 2010 and April 10, 2018 had to address any of the following: 1951 container tax, two-cents per ounce tax of 2016–2017, or tiered tax of 2018. Seventy-four articles were initially collected, of which only 49 were used in the final analysis. Excluded articles were duplicates, not set in West Virginia, failed to address the soda tax, or were subsequently removed by the publisher or online platform from a database (Figure 1). The search did not turn up letters to the editor or opinion pieces from the public. Editorial board columns were included.

#### Stage 3: Open coding of news articles

To heighten the validity of the initial coding framework two researchers individually applied open coding to read and qualitatively assess each article. This preliminary coding exercise was informed by the course materials including case studies, expert interviews, and previous studies demonstrating that the soda industry has typically positioned the SDT as a matter of individual freedom and jobs in previous policy campaigns across the U.S. Any new codes found by a researcher were added by each researcher. Differences in coding between the two researchers were discussed and resolved. Five main frames for the SDT were identified and included in the coding framework:

- Economics
- Public health concerns
- Personal liberty
- Scientific rationale
- Personal behavior

#### Stage 4: Applying the coding framework

Two researchers individually read through the articles a second time to identify appeals to authority, tax the article addressed (T1951, SDT1, SDT2), and arguments used within each of the five frames. Arguments were defined as specific elements that represent and express the underlying frame. Differences in coding were again discussed and resolved. The number of times an argument appeared in the articles was calculated (Table 2).

**Table 1:** Print News sources with online access to articles that matched our criteria

News Source	Source Location (all WV)	News Type
Bluefield Daily Telegraph	Bluefield	Newspaper
Charleston Gazette	Charleston	Newspaper
Charleston Gazette Mail	Charleston	Newspaper
Dominion Post	Morgantown	Newspaper
Fayette Tribune	Oak Hill	Newspaper
Kanawha Metro	Charleston	Newspaper
Huntington Herald Dispatch	Huntington	Newspaper
The Logan Banner	Logan	Newspaper
Montgomery Herald	Montgomery	Newspaper
Point Pleasant Register	Point Pleasant	Newspaper
Times West Virginia	Fairmont	Newspaper
Beckley Register Herald	Beckley	Newspaper
WV Metro News	Charleston	News agency (radio + online)
Associated Press	Morgantown	News agency (online)

**Figure 2a:**
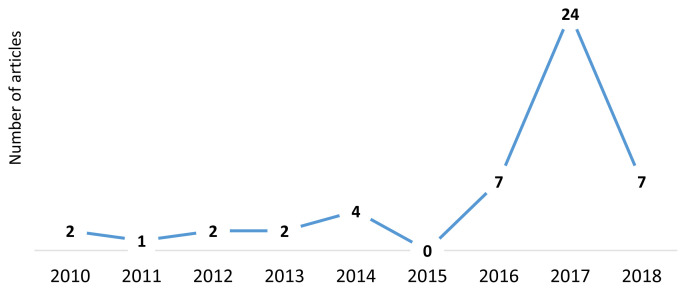
Publication year of all articles included

**Figure 2b:**
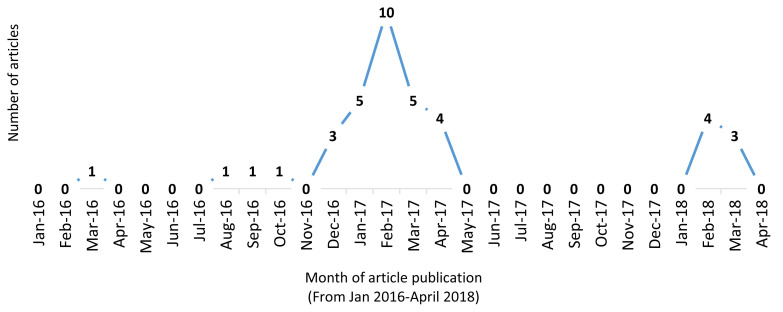
Publication (by month) of articles published from 2016 to 2018

## Supplement B

### Analysis of Frames, Arguments, and Frequency of Use

**Table 2:** Arguments and Frames

	Total	Overall % (n=49)
**Frames**		
**Arguments**		
**Economics**		
Policy will increase revenue and help balance WV budget	23	47
Policy will help fund educational programs	4	8
Policy will help fund special projects health departments, state health insurance for adults and children)	12	24
Impact of policy will be restricted to those who choose to purchase	6	12
Policy will decrease health care costs	5	10
Policy will improve workforce productivity	3	6
Policy will hurt small businesses	2	4
Policy is regressive	9	18
Policy will raise less revenue than expected	1	2
Policy will decrease the money people have to spend on other necessities	1	2
**Personal Behavior**		
Policy will lead people to choose a substitute beverage	19	39
Policy will reduce amount of SSB consumed	18	37
Policy will not change an individual’s behavior	5	10
Policy will lead people to change location of soda purchase	6	12
**Public Health**		
SSBs are a risk factor for obesity/diabetes	13	27
Policy will reduce morbidity or mortality from obesity	18	37
SSBs do not address obesity/diabetes	6	12
**Personal Liberty**		
Policy is the government’s role or responsibility	3	6
Government should not interfere	3	6
**Science Rationale**		
Sugary drinks have addictive properties	6	12
Sugary drinks have an impact of human physiology	5	10
**TOTAL**	168	

**Table 3:** Anti-tax arguments and Frames

	Total Number
**Frames and Arguments**	
**Economics**	**13**
Policy will hurt small businesses	2
Policy is regressive	9
Policy will raise less revenue than expected	1
Policy will decrease the money people have to spend on other necessities	1
**Personal Behavior**	**11**
Policy will not change an individual’s behavior	5
Policy will lead people to change location of soda purchase	6
**Public Health**	**6**
SSBs do not address obesity/diabetes	6
**Personal Liberty**	**3**
Government should not interfere	3
**Science Rationale**	**0**
**TOTAL**	**33**

**Table 4:** Pro-tax arguments and Frames

	Total Number
**Frames and Arguments**	
**Economics**	**53**
Policy will increase revenue and help balance WV budget	23
Policy will help fund special projects health departments, state health insurance for adults and children)	12
Impact of policy will be restricted to those who choose to purchase	6
Policy will decrease health care costs	5
Policy will help fund educational programs	4
Policy will improve work force productivity	3
**Personal Behavior**	**37**
Policy will lead people to choose a substitute beverage	19
Policy will reduce amount of SSB consumed	18
**Public Health**	**31**
SSBs are a risk factor for obesity/diabetes	13
Policy will reduce morbidity or mortality from obesity	18
**Science Rationale**	**11**
SSBs have addictive properties	6
SSBs have an impact on human physiology	5
**Personal Liberty**	**3**
Policy is the government’s role or responsibility	3
**TOTAL**	**135**

### Examples of Arguments for Each Frame Found in the News Articles

Economic Frame: In total there were ten types of pro and anti-tax arguments under the economic frame

Pro-tax: Economic Frame, Policy will increase revenue and help balance WV budget

*“Christine Compton, government relations director for AHA WV, said the bill would provide nearly $80 million annually to the Public Employees Insurance Agency”* – Carrie Hodousek: AHA pushes for sugary drink tax to help fund PEIA. WV Metronews 2/19/18

Anti-tax: Economic Frame, Policy is regressive

*“According to the Tax Foundation, a 10 percent soda tax could burden high-income families by $24.29, while poor families would be harmed nearly twice that amount, at $47.38. All of this adds up to an extremely bleak outlook for West Virginia’s economy, which risks the same consequences that the city of Philadelphia suffered after imposing their own beverage tax.”* – Ron Martin: Beverage tax will send consumers across state lines. Charleston Gazette Mail 4/8/17

Personal Behavior Frame: In total there were four types of pro and anti-tax arguments under the personal behavior frame.

Pro-Tax: Personal Behavior Frame; Policy will reduce amount of SSB consumed

*“The West Virginia Oral Health Coalition is in full support of the sugary drink tax, too. Together, we will be voicing our support of a modest tax that will increase the cost to consumers, in the hope that it will encourage families to choose their beverages more wisely. I've seen the results of soda in bottles and sippy cups. If mere parent education could deter excessive consumption by kids, we wouldn't need a tax. But this hasn't worked, so it's time for a bold change.”* – Dr. Vinod Miriyala: For oral health’s sake, back the sugary-drink tax. Huntington Herald Dispatch 2/21/17

Anti-Tax: Personal Behavior Frame, Policy will lead people to change location of soda purchase

*“The tax would have even greater consequences for businesses in border communities like my hometown of Bluefield, since residents will easily be able to drive over the border to shop for better prices.”* – Ron Martin:

Beverage tax will send consumers across state lines. Charleston Gazette Mail 4/8/17

Public Health Frame: The pro and anti-tax arguments had three arguments under the public health frame.

Pro-tax: Public Health Frame, Policy will make the state healthier

*“West Virginia has one of the highest obesity and diabetes rates in the nation, according to the AHA. Compton said it’s important to improve wellness in the state by decreasing the amount of sugar consumption”* – Carrie Hadousek: AHA pushes for sugary drink tax to help fund PEIA. WV Metronews 2/19/18

Anti-tax: Public Health Frame, SDTs do not address obesity/diabetes

*“We also must acknowledge what science tells us: obesity is complicated with many contributing factors. The latest data from the CDC shows that obesity rates have been going up steadily even though soda consumption has been going down*.” – Will Swann (West Virginia Beverage Association): Beverage taxes hurt working families. Huntington Herald Dispatch 2/18/18

Personal Liberty Frame: The pro and-anti-tax arguments each had one argument under the personal liberty frame.

Pro-tax: Personal Liberty Frame: Policy is the government’s role or responsibility

*“Compton called it “a choice tax.” ‘It’s a choice item. It’s not something we have to have as part of our daily diet. It’s very similar to the concept of [a] tobacco tax which did become part of the budget negotiations last year,’ she said.”* – Carrie Hodousek: American Heart Association pushes sugary drink tax in final days of legislative session. WV Metronews 4/7/17

Anti-tax: Person Liberty Frame, Government should not interfere

*“One reason that sugar-sweetened beverage, or SSB, taxes are failing is because the people are against having the money benefit government's general funds, rather than being used to prevent obesity, Caruthers said, ‘I'm here to tell you that there are a lot of people out there who do not want to grow government under any circumstance,’ he said.”* – Lori

Kersey: Taxing sugary drinks in WV advised. Charleston Gazette Mail 5/17/13

Scientific Rationale Frame: There were two pro-tax arguments and no anti-tax arguments utilized the scientific rationale frame.

Pro-Tax: Scientific Rational Frame; SSBs have addictive properties

*“That will be hard because of the addiction for one thing of the sugary drinks, as well as just learning different behaviors and choosing different options,” Drake said.”* – Carrie Hodousek: Preliminary data shows sugary drink consumption can lead to death. WV Metronews 3/26/18

**SUMMARY BOX**

**What is already known about this topic?** Media coverage of an issue and framing may influence the opinions and views of the public, decision makers, and the policy agenda.

**What is added by this report?** Similar to other regions, despite finding a predominance of pro-tax arguments, WV has had no luck in gaining legislative approval of a sugary drink tax.

**What are the implications for public health practice, policy, and research?** We recommend that future research on passage of a sugary drink tax in WV expand its focus to include other agenda setting factors such as political opportunities, governance systems, mobilizing structures and allies, and mechanisms for issue generation.
